# Implementing an inter sectoral approach to decrease the road injuries; Maragheh, Iran 

**Published:** 2019-08

**Authors:** Ali Sadeghzadeh, Sedigheh Salavati

**Affiliations:** ^*a*^Head of Traffic Police, Maragheh, Iran.; ^*b*^Maragheh University of Medical Sciences, Maragheh, Iran.

## Abstract

**Background:**

Road injuries are as one of the most important causes of deaths and premature deaths in Iran. Creating common policy dialogue and inter-sectoral collaborations are necessary in implementing the coordinated and integrated actions for preventing traffic accidents and injuries.

**Methods:**

This was an interventional study with focus on inter-sectoral collaboration approach to decrease the traffic accidents within the urban area of Maragheh city started from 2016 by Traffic Directorate in Maragheh. Main inter-sectoral traffic accidents committees in Maragheh include traffic council, traffic expert committee and traffic safety committee. Traffic injuries reduction interventions are studied, designed and implemented in these committees with participation of different stockholders.

**Results:**

Interventions to decline the rate of traffic accidents were implemented with focus on three areas of traffic management: traffic engineering, traffic culture and traffic operations between 2016 and 2018 in urban areas in Maragheh. Comparing the number of traffic accidents shows that the traffic death causalities in 2015 and 2018 was 22 and 11 deaths respectively and the number of traffic accidents leading to injuries was 935 and 697 cases respectively, so collaborative interventions resulted in decreasing deaths and injuries about 50% and 26% respectively.

**Conclusions:**

Decreasing trend in number of traffic accidents leading to injuries and deaths in recent years proves effectiveness of the inter-sectoral approach in implementing the interventions related to traffic engineering, operations and culture.

**Keywords:**

Inter sectoral approach, Traffic management, Road injuries

